# Infrared Thermography for the Ante Mortem Detection of Bruising in Horses Following Transport to a Slaughter Plant

**DOI:** 10.3389/fvets.2018.00344

**Published:** 2019-01-17

**Authors:** Rayappan Cyril Roy, Christopher B. Riley, Henrik Stryhn, Ian Dohoo, Michael S. Cockram

**Affiliations:** ^1^Department of Health Management, Atlantic Veterinary College, University of Prince Edward Island, Charlottetown, PE, Canada; ^2^School of Veterinary Sciences, Massey University, Palmerston North, New Zealand

**Keywords:** horses, transport, bruising, welfare, slaughter, digital thermography

## Abstract

Undetected injury of horses sustained during road transport to slaughter is a welfare concern. This study evaluated digital infrared thermography (DT) for the detection of ante-mortem bruising in horses following transport to a slaughter plant. The sensitivity and specificity of DT for the detection of bruises following transport was evaluated. DT images were obtained from 93 horses (2–3 horses per load; 40 loads) at a Canadian federally approved slaughter plant. From an elevated platform 5 m from the horses, left and right lateral DT images, and one caudal pelvic area image were obtained from each horse. After slaughter the carcasses were examined for bruising (a visually discolored area on the carcass caused by damage to the blood vessels) and findings documented. Sensitivity, specificity, and predictive values were calculated for DT assessment of bruising. The prevalence of bruising on post mortem inspection was 54%. The DT approach to bruise detection at the region of interest level of 93 horses (*n* = 186 sides) resulted in a sensitivity of 42% and specificity of 79%. As the sensitivity was low, a more sensitive DT camera and allowing for a longer equilibration time for horses after transport may improve this approach to post transport assessment of subclinical injury.

## Introduction

The transport of horses for slaughter and related management practices may cause externally visible and non-visible injuries due to trauma including fractures, swelling, excoriations, and bruising (1, 2). The welfare assessments of livestock undertaken following transport to slaughter plants include visual/clinical evaluations by plant personnel or official inspectors (3). However, horses may have non-visible injuries unrecognized until post-mortem carcass examination (1). The lack of suitable methods to identify bruised horses ante-mortem limits the ability to differentiate injuries occurring during transport, from those that occur at the slaughter plant itself (4). It is important to determine which stage of the process is responsible for bruising, so that appropriately directed measures are taken to reduce this risk. Reliable and objective tools to detect non-visible injuries in horses following transport could empower regulatory authorities and plant operators to improve animal transport welfare (5, 6).

Digital thermography (DT) is a non-invasive imaging technique that records superficial infrared emission patterns (7). When tissue is damaged, localized hypo- or hyper-perfusion due to vascular injury and inflammation occurs that may be detectable by DT (8, 9). In horses, DT has been used to detect musculoskeletal and neuromuscular injuries, and to monitor skin lesions (7, 10, 11). It also has been used for the ante mortem and post mortem detection of blunt force trauma in humans (12, 13). The authors hypothesized that a qualitative methodology using DT imaging implemented at slaughter plants may identify horses with bruising ante-mortem after transport. The objective of this study was to estimate the sensitivity and specificity of DT images as a diagnostic test for detecting bruising ante-mortem when compared to post-mortem visual examination of carcasses.

## Materials and Methods

Ethical approval for this study was granted by the University of Prince Edward Island (UPEI) Animal Care Committee (Animal Care Protocol #12-003). The study was evaluated and approved by the UPEI Institutional Biosafety Committee (Bio Hazard Protocol #6003835).

### Capture of Ante Mortem Digital Thermography Images

A thermography camera (Model i7, FLIR Systems, Inc. 27700 SW Parkway Ave, Wilsonville OR 97070, USA) was used to obtain DT images. The camera was calibrated so that those areas on the skin surface of each the horse with a temperature higher than a threshold temperature appeared as red areas on the DT image. A threshold temperature was determined within each horse to account for intrinsic physiological differences contributing to variation in baseline thermal measurements (5, 14, 15). The threshold temperature was calculated by obtaining spot surface temperature measurements for each horse at three locations on the neck, dorsal pelvic, and flank regions at a distance of 5 m (Supplementary Figure 1). The three locations chosen were based on the approach described by Soroko et al. (16) and a pilot study conducted by the authors of skin temperature measurement by DT of healthy horses outdoors concluding that temperature readings were more stable at these three locations (17). The three spot temperatures were averaged and the value entered into the camera as the threshold surface temperature for that particular horse to detect thermal anomalies. Emissivity was set at 0.96 for all DT images. The distance selected was based on the operational requirements at the slaughterhouse that required the observer to be at a safe distance from the horses at all times.

Following the calibration of the DT camera, horses were imaged on arrival at the lairage (an unheated open barn-like area comprised of a roof with the upper third of the side walls open to the environment on all four sides) of a federally approved slaughter plant in Quebec, Canada. A convenience sample of 93 adult horses (2–3 horses chosen from 40 truckloads) were selected. The measured environmental temperature closely followed the measured ambient outdoor temperature. From an elevated platform 5 m away, one left and one right lateral body image and one caudal view of the pelvic area were obtained for each horse. To ensure the correct identification of horses with post-mortem carcass bruising, images were only obtained from those that were moved immediately to slaughter after unloading from the transport vehicle.

### Post-mortem Evaluation of Horse Carcasses

After humane slaughter and hide removal, all carcasses were visually examined for bruising by one observer (RCR), and the findings were recorded using anatomical landmarks on pre-printed horse outline diagrams, representing the left and right sides of the horse. A bruise on the carcass was defined as a visually discolored superficial area on the carcass caused by damage to the subcutaneous blood vessels. No distinction was made between bruises that were superficial and those that may have affected deeper tissues. Determination of the latter would have required the operators to permit dissection of the carcasses. Bruising was assessed immediately after slaughter using a modified subjective scoring system based on an adaptation of the Australian Carcass Bruise Scoring System for cattle, accounting for other possible discolorations (18, 19).

### Analysis of Digital Thermography Images

All DT images were downloaded for assessment using ThermaCAM Researcher software (FLIR Systems, Inc. 27700 SW Parkway Ave, Wilsonville OR 97070, USA). Not all regions of elevated thermal emission were considered as potential bruising; some are associated with anatomical structures where the blood supply is normally greater than in the surrounding areas (9). To eliminate regions of physiologically increased emission from those potentially associated with pathological surface temperature increase (i.e., possible bruising), the left and right lateral side images were compared. Thermal regions of interest that were asymmetric in only one image corresponding to one side of the body were considered as potentially bruised. To refine DT identification of bruising in the lateral regions, the pelvic image was used to mitigate the effect of the angle at which the lateral image was obtained. Regions of elevated thermal emission potentially representing bruising were marked on two horse outline diagrams (representing the left and right sides) so that comparisons could be made with the outline diagrams of actual bruising identified from carcasses. Therefore, for each horse an outline diagram indicating one side (left or right) of a horse was the unit of measurement (*n* = 186 sides). If an asymmetric thermal region of interest anatomically coincided with a carcass bruise marking, then that horse outline diagram (representing one side of the horse) was considered positive for bruising (true positive). When DT identified one positive bruise, other symmetric or asymmetric zones of increased thermal emission patches in the same horse outline diagram were not further considered, thereby reducing the unit of measurement to two units per horse (left and right side). If there were one or more asymmetrical regions of thermal emission without corresponding bruising from the carcass assessment, then that diagram was considered a false positive. Outline diagrams with no bruise on the carcass and no thermal asymmetry on DT were considered true negatives; those showing a bruised carcass but no thermal asymmetry on DT were considered false negatives.

### Data Analysis

The threshold temperature measurements were converted from the Celsius to Kelvin units to allow for computation of the coefficients of variation, and the means for each animal plotted to evaluate the precision of the threshold temperature as a reference for the detection of bruising. The post mortem carcass assessment for bruising was used as the reference test against which DT image based assessments were compared. Sensitivity, specificity, and positive and negative predictive values were calculated for the qualitative DT assessment test for bruising against carcass inspection as the reference test.

## Results

**Figure 1:**
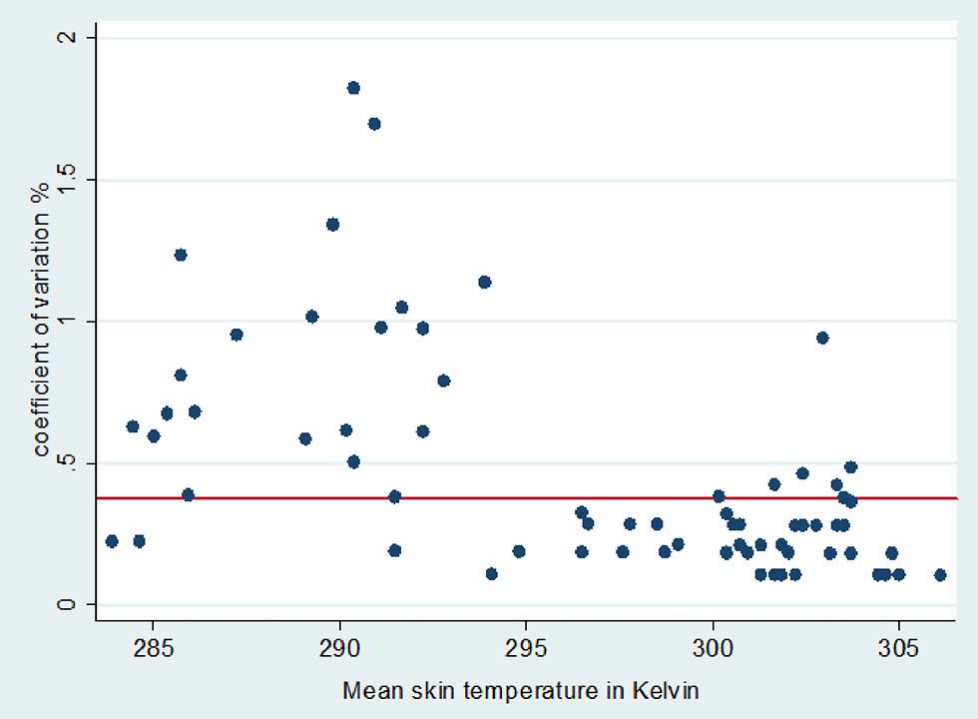
Coefficient of variation (%) plotted against threshold temperature in Kelvin (threshold temperature was the mean of three spot skin temperature at neck, pelvis and flank). The horizontal line indicates the mean skin temperature coefficient of variation for all horses examined.

**Figure 2:**
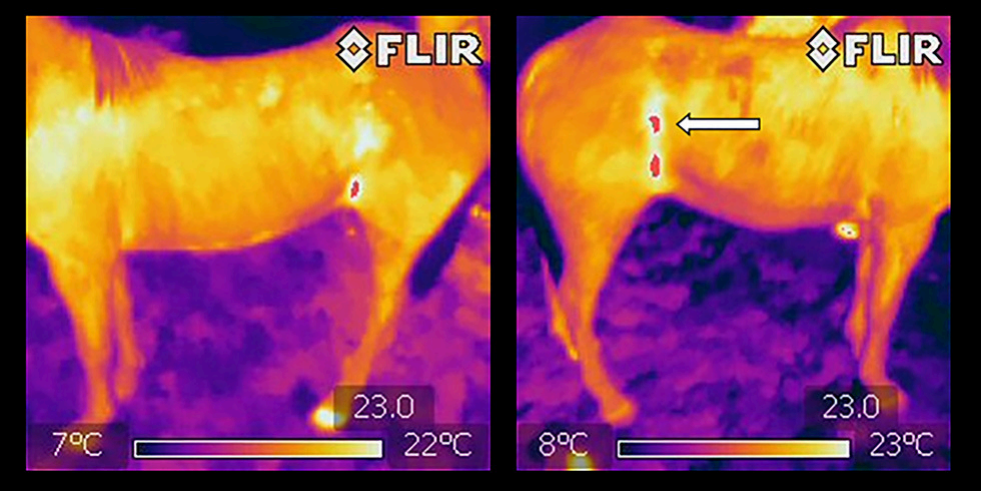
Left lateral and right lateral digital thermography images of the same horse acquired at a distance of 5 m to detect potential bruising. Arrow in the right lateral image shows an asymmetric high temperature zone (red “patch”) when compared to left lateral view.

The prevalence of bruising determined by carcass inspections was 54% (100 of 186 sides), and 33% (61 of 186 sides) had observable skin injures. The average threshold temperature was 25.0 ± 6.2°C, and the coefficient of variation values for each horse were closely dispersed about the mean coefficient of variation (Figure 1). Figure 2 (a and b) illustrates DT images of a horse with suspected ante mortem bruising in the right flank region. Comparisons between the visual carcass inspection and DT methods of bruise identification at the region of interest level (left and right sides) of 93 horses (*n* = 186) produced a sensitivity of 42% and specificity of 79% at a prevalence of 54%. The corresponding positive predictive value of the DT approach to bruise detection was 84% and the negative predictive value was 35%.

## Discussion

Information on the prevalence of bruising following transportation of horses prior to slaughter is limited. However, the value reported in the USA by one group of workers (51%) (1) and a second for adult horses in Iceland (58%) (19) closely approximates findings in the current study. In this study, a qualitative ante-mortem approach using DT sought to assess bruising by identifying body parts with elevated skin temperatures. Thermal symmetry served as an additional measure to improve the specificity to detect bruising, although this may reduce sensitivity (7). Asymmetry in skin temperature has been used diagnostically for lameness in dairy cattle and horses (20, 21), and human breast cancer (22). These studies suggest that within animal controls can be used to detect pathology, but a temperature difference > 1.25°C is required in the horse (7). Using thermal symmetry in the current study resulted in a relatively modest specificity of 79% for bruise detection compared to 95% in the detection of equine lameness (21).

Choosing a threshold temperature is an important step when using DT qualitatively under outdoor conditions. Under indoor conditions in thermo-neutral zone normal skin temperatures are ~5°C cooler than core values, hence 32°C may be chosen as the threshold temperature (23). However, since this study was undertaken in the partially sheltered conditions of the slaughterhouse lairage, the skin temperature of horses are more likely to have differed according to the ambient temperature (7, 20). Hence, in similar imaging environments, a flexible threshold temperature must be established for each examination to detect thermal anomalies that may be indicative of bruising or other pathology. Although not observed in the current study, possible bruising at the sites used to establish a threshold temperature may increase the number of false positives detected. However, in the current study the coefficient of variation graph indicates that the variability of threshold temperature used for each animal was acceptable, and the use of three spot temperature points to establish a threshold resulted in sufficient precision in most horses for DT under field conditions where indoor assessment is not possible.

Ideally, animal inspectors or welfare assessors evaluating the welfare status of horses following transport (the pre-slaughter stage) require a simple diagnostic tool to identify horses with bruising using a practical screening test. To achieve this objective, the DT images should have high sensitivity thereby detecting all bruised horses. However, in this study the qualitative diagnostic test to detect bruising in horses had low sensitivity, a finding that was consistent with those of others that have used DT qualitatively (7, 22). This may in part have been associated with the distance between the horse and detector; with increasing distance a decrease in resolution may occur with fewer pixels in the area of interest (24). A previous limited pilot study by the authors did not find a significant change in sensitivity over a distance of 1–6 m, but a more thorough investigation is warranted (17, 24).

Although the qualitative diagnostic approach using DT to detect bruising had a high positive predictive value, predictive values are highly influenced by the prevalence of disease or injury. Perhaps more importantly, many animals later found to be bruised at slaughter may not be detected shortly after transport. In the current study horses were evaluated shortly after disembarking from transport, and there may have been insufficient equilibration time for the inflammatory response to injuries sustained during transport to overcome the contributions of thermal emission by the skin and subcutaneous tissue (9). Although inflammation commences shortly after tissue injury, inflammation may take 1–3 days to peak in the horse, and revascularization may not commence for 3–5 days (25). Early inflammatory changes may require more sensitive methods for detection (12). One possible way to reduce the number of false negatives includes reducing the preset threshold temperature value thereby increasing the sensitivity, with a possible loss in specificity. A second approach is to provide a greater equilibration time for horses after transportation before DT images are obtained, and to give them time to recover from the effects of transport (5, 5, 14). In the current study, horses sent for slaughter immediately after unloading were purposefully chosen for the study to facilitate follow up of the same animal after stunning for carcass examination.

Plausible reasons for false positives observed were skin wounds (e.g., abrasions) without bruising, physical contact with other horses increasing surface temperature, and hyperemic cutaneous tissues that were otherwise not sufficiently traumatized to cause muscle bruising (15). As noted in the results, a significant percentage of horses in this population had skin trauma. Contact with the vehicle can also increase or decrease skin temperature of an animal in specific locations because of contact with the metal body of the vehicle. For example, this was evident from the DT images of a few sampled uncastrated horses that were transported with partitions. Tuber coxae of these horses in contact with partitions had elevated skin temperatures, but no post-mortem evidence of bruising.

In conclusion, the methodology used here to detect bruising ante-mortem had a modest sensitivity for bruise detection and good specificity. Increasing the equilibration time provided for the horses after transport and controlling the ambient temperature within the lairage in the thermo-neutral range for horses may increase the sensitivity of this approach.

## Author Contributions

RR and MC conceived the study. RR, MC, and CR contributed to development of the methodology. RR collected the data. RR, HS, and ID performed the data analysis. RR and CR wrote sections of the manuscript. All authors contributed to manuscript revision, read and approved the submitted version.

### Conflict of Interest Statement

The authors declare that the research was conducted in the absence of any commercial or financial relationships that could be construed as a potential conflict of interest.

## References

[1] Grandin T McGee K Lanier JL. Prevalence of severe welfare problems in horses that arrive at slaughter plants. J Am Vet Med Assoc. (1999) 214:1531–3. 10340083

[2] Marlin D Kettlewell P Parkin T Kennedy M Broom D Wood J. Welfare and health of horses transported for slaughter within the European Union Part 1: Methodology and descriptive data. Equine Vet J. (2011) 43:78–87. 10.1111/j.2042-3306.2010.00124.x 21143638

[3] Grandin T. Auditing animal welfare at slaughter plants. Meat Sci. (2010) 86:56–65. 10.1016/j.meatsci.2010.04.022 20599326

[4] Sandström V Development of a Monitoring System for the Assessment of Cattle Welfare in Abattoirs. Student report 293 Swedish University of Agricultural Sciences. (2009). Available online at: http://citeseerx.ist.psu.edu/viewdoc/download?doi=10.1.1.571.9581&rep=rep1&type=pdf

[5] Gloster J Ebert K Gubbins S Bashiruddin J Paton DJ Normal variation in thermal radiated temperature in cattle: implications for foot-and-mouth disease detection. BMC Vet Res. (2011) 7:73–82. 10.1186/1746-6148-7-73 PMC3235061 22104039

[6] Rainwater-Lovett K Pacheco JM Packer C Rodriguez LL. Detection of foot-and-mouth disease virus infected cattle using infrared thermography. Vet J. (2009) 180:317–24. 10.1016/j.tvjl.2008.01.003 18308596 PMC7110760

[7] Soroko M Howell K Infrared thermography: current applications in equine medicine. J Equine Vet Sci. (2018) 60:90–6. 10.1016/j.jevs.2016.11.002

[8] Yanmaz LE Okumus Z Dogan E Instrumentation of thermography and its applications in horses. J Anim Vet Adv. (2007) 6:858–62.

[9] Soroko M Davies Morel MGG Equine Thermography in Practice. Oxfordshire: CABI (2016).

[10] Braverman Y Potential of infrared thermography for the detection of summer seasonal recurrent dermatitis (sweet itch) in horses. Vet Rec. (1989) 125:372–4. 10.1136/vr.125.14.372 2815515

[11] Celeste CJ Deschesne K Riley CB Theoret CL Skin temperature during cutaneous wound healing in an equine model of cutaneous fibro proliferative disorder: kinetics and anatomic-site difference. Vet Surg. (2013) 42:147–53. 10.1111/j.1532-950X.2012.00966.x 22742866

[12] Bariciak ED Plint AC Gaboury I Bennett S. Dating of bruises in children: an assessment of physician accuracy. Paediatrics (2003) 112:804–7. 10.1542/peds.112.4.804 14523170

[13] Bernstein M Nichols G Blair J. The use of black and white infrared photography for recording blunt force injury. Clin Anat. (2013) 26:339–46. 10.1002/ca.22078 22648741

[14] Tunley BV Henson FMD. Reliability and repeatability of thermographic examination and the normal thermographic image of the thoracolumbar region in the horse. Equine Vet J. (2004) 36:306–12. 10.2746/0425164044890652 15163036

[15] Autio E Neste R Airaksinen S Heiskanen M. Measuring the heat loss in horses in different seasons by infrared thermography. J Appl Anim Welf Sci. (2006) 9:211–1. 10.1207/s15327604jaws0903_3 17112332

[16] Soroko M Dudek K Jodkowska E Henklewski R Thermographic evaluation of racehorse performance. J Equine Vet Sci. (2014) 34:1076–83. 10.1016/j.jevs.2014.06.009

[17] Roy RC Welfare of Horses Transported to Slaughter in Canada Iceland: Assessment of Welfare Issues Associated Risk Factors. Doctoral thesis, University of Prince Edward Island, Charlottetown, PE. (2014). Available online at https://www.islandscholar.ca/download_ds/ir:12190/OBJ/ir_12190.pdf

[18] Anderson B Horder JC The Australian carcase bruise scoring system. Queensland Agric J. (1979) 105:281–7.

[19] Roy RC Cockram MS Dohoo IR Ragnarsson S Transport of horses for slaughter in Iceland. Anim Welf. (2015) 24:485–95. 10.7120/09627286.24.4.485

[20] Alsaaod M Büscher W. Detection of hoof lesions using digital infrared thermography in dairy cows. J Dairy Sci. (2012) 95:735–42. 10.3168/jds.2011-4762 22281338

[21] Soroko M Henklewski R Filipowski H Jodkowska E The effectiveness of thermographic analysis in equine orthopaedics. J Equine Vet Sci. (2013) 33:760–2. 10.1016/j.jevs.2012.11.009

[22] Kontos M Wilson R Fentiman I. Digital infrared thermal imaging (DITI) of breast lesions: sensitivity and specificity of detection of primary breast cancers. Clin Radiol. (2011) 66:536–9. 10.1016/j.crad.2011.01.009 21377664

[23] Turner TA. Diagnostic thermography. Vet Clin North Am Food Anim Pract. (2001) 17:95–113. 10.1016/S0749-0739(17)30077-9 11488048

[24] Westerman S Buchner HH Shramel JP Tichy A Stanek C Effects of infrared camera angle and distance on measurement and reproducibility of thermographic determined temperatures of the distal lateral aspects of the forelimbs in horses. J Am Vet Med Assoc. (2013) 242:388–95. 10.2460/javma.242.3.388 23327183

[25] Theoret C Physiology of wound healing. In: Theoret C Schumacher J editors. Equine Wound Management. 3rd ed Ames, IA: John Wiley & Sons Inc (2017). p. 1–13.

