# Supplementary figures and images for: Infrared Thermography for the Ante Mortem Detection of Bruising in Horses Following Transport to a Slaughter Plant
Source: Front Vet Sci. 2019 Jan 17;5:344. doi: 10.3389/fvets.2018.00344 (PMC6344440; doi:10.3389/fvets.2018.00344)

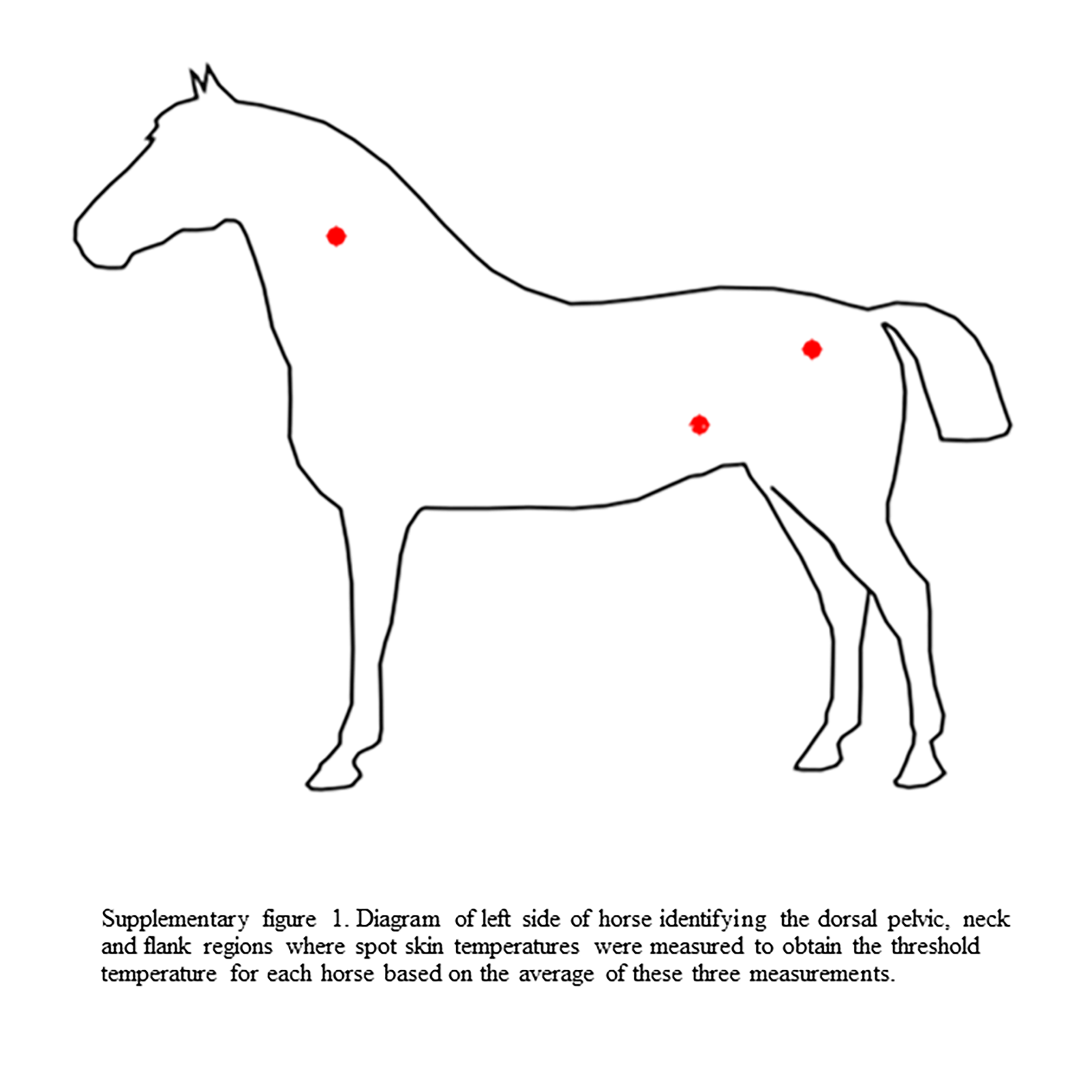

Supplement: Supplementary file 1 [file Image_1.TIF]
